# Social contact networks for the spread of pandemic influenza in children and teenagers

**DOI:** 10.1186/1471-2458-8-61

**Published:** 2008-02-14

**Authors:** Laura M Glass, Robert J Glass

**Affiliations:** 1 Albuquerque Public Schools, Albuquerque, New Mexico, USA; 2 National Infrastructure Simulation and Analysis Center (NISAC) [NISAC is a program of the Department of Homeland Security's Infrastructure Protection/Risk Management Division and comprised of a core partnership of Sandia National Laboratories (SNL) and Los Alamos National Laboratory (LANL)] Albuquerque, New Mexico, USA; 3 Sandia National Laboratories (SNL) [SNL is a multiprogram laboratory operated by Sandia Corporation, a Lockheed Martin Company for the United States Department of Energy's National Nuclear Security Administration under contract DE-AC04-94AL85000] Albuquerque, New Mexico, USA

## Abstract

**Background:**

Influenza is a viral infection that primarily spreads via fluid droplets from an infected person's coughs and sneezes to others nearby. Social contact networks and the way people interact within them are thus important to its spread. We developed a method to characterize the social contact network for the potential transmission of influenza and then applied the method to school aged children and teenagers.

**Methods:**

Surveys were administered to students in an elementary, middle and high-school in the United States. The social contact network of a person was conceptualized as a set of groups to which they belong (e.g., households, classes, clubs) each composed of a sub-network of primary links representing the individuals within each group that they contact. The size of the group, number of primary links, time spent in the group, and level of contact along each primary link (near, talking, touching, or kissing) were characterized. Public activities done by groups venturing into the community where random contacts occur (e.g., friends viewing a movie) also were characterized.

**Results:**

Students, groups and public activities were highly heterogeneous. Groups with high potential for the transmission of influenza were households, school classes, friends, and sports; households decreased and friends and sports increased in importance with grade level. Individual public activity events (such as dances) were also important but lost their importance when averaged over time. Random contacts, primarily in school passing periods, were numerous but had much lower transmission potential compared to those with primary links within groups. Students are highly assortative, interacting mainly within age class. A small number of individual students are identified as likely "super-spreaders".

**Conclusion:**

High-school students may form the local transmission backbone of the next pandemic. Closing schools and keeping students at home during a pandemic would remove the transmission potential within these ages and could be effective at thwarting its spread within a community. Social contact networks characterized as groups and public activities with the time, level of contact and primary links within each, yields a comprehensive view, which if extended to all ages, would allow design of effective community containment for pandemic influenza.

## Background

The spread of infectious diseases in today's highly connected world is one of public health's most important problems. Recently, a number of studies have applied computational models to understand the spread and evaluate the containment of pandemic influenza both from a source in south-east Asia [1,2] and within the United States and Great Britain [3,4]. However, in these models, influenza spreads within communities through random contacts within fully mixed groups of various sizes rather than along true social contact networks that reflect the cliquish nature of people's interactions. Thus, the design of community containment and practice based on such model results is not yet robust [5].

Influenza is a viral infection that primarily spreads when an infected person coughs or sneezes fluid droplets containing a virus that another person may come into contact with through the air and sometimes on surfaces. Most transmission of influenza probably happens within 3 feet of the source – the coughing infected person [6,7]. Thus, the social contact network, and the way people interact within, is critical to the spread of influenza. Computational models that mimic true social contact networks would both reduce the uncertainty of results that simulate influenza's spread and help design robust measures to best stop or contain it at the community level. Social contact networks representative of stylized towns in the United States have recently been embodied within computer simulations [8,9]. These studies have demonstrated that social distancing measures can be designed to target the portion of the social contact network most responsible for influenza's spread and thus guide the design of effective community measures.

Critically important for influenza's transmission are school aged children and teenagers [8-13]. Thus to understand influenza's spread and how to best contain a pandemic locally, the social contact network of children and teenagers must be understood and characterized. To date, little research has focused on characterizing this important portion of the social contact network for the spread of influenza [14]. Are there critical groups or activities as well as critical people in its spread, and does this change with age? How important are random connections as occur in passing within shopping malls or schools? Can social distancing, aimed at critical groups and age classes successfully reduce transmission and locally thwart a pandemic? In this paper, we developed an approach to characterize social contact networks for the potential transmission of influenza in school aged children and teenagers. We then applied the approach in Albuquerque, NM, USA, a medium sized (population ~500,000) suburban western city, and analyzed the results to understand the basic features of the student's social contact networks, the critical groups and public activities for transmission, the relative importance of random contacts, and the tendency of like age to interact with like (assortativity). In light of these data, we analyze the community containment strategy of closing schools and keeping students at home on the potential of transmission within children and teenagers during a time of influenza pandemic.

## Methods

In their model, Glass et al. [8,9] conceptualize a social contact network as people of appropriate ages placed into multiple groups (more than one group per person) of a variety of types such as households, school classes, or clubs (see Additional file 1, Figure S12 for an illustration). Within each group, people are connected to each other with primary links (networked as a variety of random to regular to scale-free graphs) to yield cliques or seating (as in a classroom) such that an individual does not necessarily have contact with all others in the group. Because people belong to multiple groups, the social contact network exhibits the overlapping quality of a structured community [15] with both clustering within a group (cliques) and the "small-world characteristic" of needing only a small number of contacts along links to reach any particular individual within the entire community [16]. The network is generated from statistics (averages and distributions) for the groups people belong to, their size, internal structure such as seating, the number of primary links that a person may have within each group, and the frequency of contact along these links. The frequency of contact is related to the amount of time that a person spends within a given group and may vary from group to group and person to person.

Three recent studies consider the contact process within a social network to evaluate the spread of infectious diseases such as influenza [14,17,18]. Using surveys and contact diaries they were able to show a direct connection between observations of age-specific social behavior and the observed age-specific risk of infection. Such studies support the use of self-reported social contacts as a way to evaluate the transmission of infection, as well as do others in the characterization of more general social networks [19,20]. Of particular interest, Edmunds et al. [17] differentiated contacts into 4 levels: level 1 for physical contact without conversation, level 2 for conversation, level 3 for conversation with physical contact, and level 4 for kissing and other intimate behavior. These levels relate to the potential that an infection like influenza will be passed during a contact.

We combined Edmunds et al.'s [17] concept of contact-level with Glass et al.'s [8,9] conceptualization of a social contact network to obtain a group-centric method of characterization that embodied both quantity (time) and quality (level) of contact for the potential transmission of influenza. We initially evaluated two self-report methods, surveys of individuals and contact diaries. Surveys of individuals produce data based on a person's reflection and estimation of what they do in their lives on typical days, weeks, or longer times, and if non-specific prompting is used, forgetting can be counteracted at least in part [21]. Contact diaries in principle collect actual contact data from individuals over a specific period of time as the contact occurs. In practice, we found in preliminary trials that students filled in their contact diaries very unevenly and often from memory well after the contact (through reflection and estimation) rather than at the time of the contact. Therefore, we chose to use individual surveys conducted at once with entire school classes, that is, students were guided to fill out the survey individually (without comparison) but in class, step by step, with instruction and examples given as required. This gave access to students in a structured educational setting with an authority figure present (teacher) and where questions could be asked and answered with non-specific prompting. Our survey questionnaire and its administration within a classroom setting are provided in Additional file 1.

In the survey, students recorded what they do on typical school days, weekends, weeks, months or years as relevant for a particular group or "public activity" (see definition Table 1). The distinction between groups and public activities is critical for our characterization. Groups are defined as routine sets of people within which a person contacts others and were categorized as households, extended family, before school classes or care, school classes, lunch periods and recess, after school care, clubs, sports, work, church, friends, and neighborhood. Public activities were called out separately and defined as activities that are usually done either alone or as part of a group venturing out into the community where many unplanned or random interactions can occur. These public activities were categorized as passing periods within school, car rides, school and city bus rides, mall, errands, movies, concerts, sport event participation or attendance, dances, parties, and eating out.

**Table 1:** Definitions

**General Terms**	
**Term**	**Definition**

Groups	Routine sets of people within which a person contacts others. School age children and teenagers belong to a set of group types that we categorized as households, extended family, before school classes or care, school classes, lunch periods, recess, after school care, clubs, sports, work, church, friends, and neighborhood.
Public Activities	Activities done either alone or as part of a group venturing out into the community where many unplanned or random contacts can occur. Public activities were categorized as passing periods within school, car rides, school and city bus rides, mall, errands, movies, concerts, sport event participation or attendance, dances, parties, and eating out.
Contact	An interaction with another person during which influenza could be passed. These must be within 3 ft and for a recognizable length of time. Contacts are divided into two types, those that occur within Groups and those that occur during Public Activities. Contacts in groups were with primary links in groups and took place for significant time, more than several minutes (often hours). Contacts in Public Activities were with primary links in the group that is doing the activity and random contacts with others (not the primary links within the group). Random contacts were for much less time than those with primary links in groups (tens of seconds to several minutes).
Contact level	Because not all contacts may be equal with respect to the spread of an infectious disease such as influenza, we defined four contact levels (all must be within 3 ft and for a recognizable length of time): 1: close, within 3 feet 2: close and talking 3: close, talking and touching 4: kissing
Primary links in groups	Within a group, a person's primary links are other people in the group that they are within 3 ft significant time, more than several minutes (often hours). For example, in a school class, a student may be in a group with 30 other students but may only be within 3 ft with the 4 people seated around them, thus the size of group would be 30 and number of primary links would be 4. Conversely, in a friend group, the student may have primary links with all others within that group.
Random contacts	Unplanned contacts where influenza could be passed that occur in public activities in public places such as school hallways, malls, movie theatres, etc. These also occur in the work environment with "customers". Random contacts are not with primary links in the group that is doing the public activity (or work). Random contacts must be within 3 ft and for a recognizable amount of time but less than a few minutes. Data on the amount of time associated with random contacts was not taken, they were defined as "in passing" (less than a few minutes, with other students in hallways and people in malls, concerts, dances, etc, or customers in the work environment).
**Group Measures**	
Number Observations (Num Obs)	Number of groups of a particular type totaled across all individuals for the given grade (or grade range).
Number Groups per person	Average & CV for number of groups of a particular type that an average person has for the given grade (or grade range). The number of households per person can be greater than one if there are students that live at more than one household (often divorced or separated parents).
Size	Average & CV for the number of people in groups of a particular type for the given grade (or grade range).
Time per day	Average & CV for time spent in a group of a particular type per day for the given grade (or grade range).
Primary Links	Average & CV for number of primary links a person has in a group of a particular type for the given grade (or grade range).
Contact-hours per day	Average & CV for number of contact-hours a person has in a group of a particular type per day (number of primary links multiplied by the time in the group per day) for the given grade (or grade range).
Contact-level	Average & CV for contact-level of primary links in a group of a given group type for the given grade (or grade range).
Contact-level-hours per person per day	Average & CV for the number of contact-level-hours in a group of a particular type that an average person has per day for the given grade (or grade range). (Contact-hours per day for a particular group multiplied by the average contact-level for the group are first averaged for each group type. This value for each group type is then multiplied by the number of groups of this type per person.)
**Public Activity Measures**	
Number of Observations (Num Obs)	Number of public activities of a particular type totaled across all individuals in the given grade (or grade range).
Number of Activities per person per day	Average & CV for number of public activities of a particular type that an average person has per day for the given grade (or grade range).
Time per participation	Average & CV for amount of time spent doing a particular public activity once for the given grade (or grade range).
Primary links	Average & CV for number of primary links from the group that a student does the particular public activity with for the given grade (or grade range).
Contact-hours per participation	Average & CV for number of contact-hours (primary links multiplied by time per activity) for a particular public activity done once for the given grade (or grade range).
Contact-level	Average & CV for contact-level of primary links within the group doing a public activity of this type for the given grade (or grade range).
Contact-level-hours per person per day	Average & CV for the number of contact-level-hours in a particular type of public activity that an average person has per day for the given grade (or grade range). (Contact-hours per activity for a particular activity multiplied by the average contact-level with primary links for the group the activity is done with is first averaged for each activity type. This value for each activity type is then multiplied by the number of activities of this type per person per day.)
Participating group	Group(s) that students recorded doing a public activity with. H stands for household, f for friends, and sports for sports group.
Fraction counted in groups	Fraction of students in a given grade (or grade range) who reported the time spent in a particular public activity as already counted in the time they spent with the participating group.
Random contacts per participation	Average & CV for number of random contacts a student has while participating in a particular public activity once for a given grade (or grade range).
Random contacts per person per day	Average & CV for number of total random contacts for each student in a particular public activity on a per-day basis for a given grade (or grade range).
Random contacts contact-level	Average & CV for contact-level reported for random contacts by students in a particular public activity for a given grade (or grade range).
**Other terms**	
Per-person	Per-person values were average values for the particular grade (or grade range).
Per-day	Per-day values were formed for an average day that incorporated both week days and weekends (so that some weekday time such as in school classes was distributed to the weekend and vice versa for extra time spent on weekends with household members).

Students first filled in their age, grade and gender and then proceeded to fill in a set of tables which assessed the groups (listed above) and group characteristics to which they belonged. Group characteristics (defined in Table 1) included: group name, time in group per day or week, group size, age range of entire group, number of primary links inside the group (within 3 ft and for a significant amount of time, more than several minutes; for example, a student may be in a school class group with 30 other students but may only be within 3 ft of and interact with the 4 people seated around them, thus the size of group would be 30 and number of primary links would be 4), initials of primary links, relationship with primary links (family, friend, acquaintance, authority), contact-level or range with primary links (1: close, within 3 feet, 2: close and talking, 3: close, talking and touching, 4: kissing), and ages or age range of primary links. While not used in subsequent analysis, filling in the initials and relationship of their primary links prompted students to focus and carefully think through their groups and contacts throughout the day. Such non-specific prompting was found in preliminary trials to be important to increase survey accuracy as has also been found more generally by others [21]. Students were also asked to record additional time spent with household members on the weekend and the number of hours they spent alone (doing homework, watching TV, reading, etc.) on a daily basis. The hours a student spent sleeping were not recorded and thus excluded from attribution. In the work environment, random contacts (defined as "customers") were also recorded.

After completing the groups section of the survey questionnaire, students then filled in a set of tables to characterize the public activities (listed above) in which they participated. Characteristics of public activities (defined in Table 1) were: number of times per day, week or month that an individual participates in the public activity, how much time the public activity takes, which previously defined groups the individual does the public activity with, number of the primary links within these groups an individual has contact with during the activity, range of level of these contacts, whether the public activity is already included in the time spent previously recorded in the groups, and the number, range of level of contact and age of others contacted at random while doing the activity. Data on the amount of time associated with random contacts was not taken, they were defined as recognizable but "in passing" (less than a few minutes, with other students in hallways and people in malls, concerts, dances, etc, or customers in the work environment). Surveys often required more than one class period to complete.

In-class surveys were conducted during late fall, 2006, and early winter, 2007, after receiving study approval from the Albuquerque Public School Review and Clearance Committee. Albuquerque High School and one of its feeder middle and elementary schools were chosen as they were both ethnically and socio-economically diverse. A convenience sample was chosen of 2 classes of freshmen (9^th ^grade, ages 14–15), sophomores (10^th ^grade, ages 15–16), juniors (11^th ^grade, 16–17) and seniors (12^th ^grade, ages 17–18), as well as 2 middle school classes (7^th ^grade, ages 12–13) and 2 elementary school classes (5^th ^grade, ages 10–12) for the survey (a total of 12 classes and 249 people). Younger children were not considered because of the difficulty of completing the survey questionnaire. Out of 249 survey questionnaires administered, 141 (57%) were used for subsequent analysis (legible and fully filled in) with 82 from female and 59 from male students.

Data from surveys were sorted by grade (bunching 9–10^th ^and 11–12^th ^grades together) and by group/public activity type and then statistics were calculated (averages, standard deviations (SD), coefficient of variation (CV = SD/Average), minimum, and maximum values) for each group/public activity for the following measures (defined in Table 1): number per person, size, time (given for groups per day; for public activity per participation in the activity), number of primary links, contact-hours (number of primary links multiplied by time), contact-level, contact-level-hours (contact-level multiplied by contact-hours) and contact-level-hours per-person-per-day. Per-person values were calculated using the average number of the particular group/public activity for students in the particular grade (or grade range). For example, students in 11–12^th ^grade had on average 1.52 different groups of friends each with an average of 16.18 contact-level-hours per day thus yielding 24.59 contact-level-hours per-person-per-day. Per-day values were formed for an average day that incorporated both week days and weekends (so that some weekday time such as in school classes was distributed to the weekend and vice versa for extra time spent on weekends with household members).

Contact-level-hours per-person-per-day combines both the quality and quantity of contact and is our best estimation of potential influenza transmission within a group or public activity. However, at its base, it is formed by a simple product between the level of the primary link and the time for the link within the group. In this way, level 2 is presumed to have twice the potential of transmission of level 1, etc. While this has the correct trend, it is a simple linear approximation.

Data for individual students were also analyzed to consider the total number of groups they belonged to, and, for their groups/public activities, the total contact-hours per day, average contact-level, and total contact-level-hours per-day. Across all individuals, we also calculated the fraction of primary links and random contacts that were with each of 5 age classes: preschoolers (0–5 years), elementary school children (6–10 years), middle-high school teenagers (11–19), adults (20–64), and seniors (>65). Finally, the average number of random contacts and their level in each of the public activities and the work environment were calculated.

## Results

**Figure 1:**
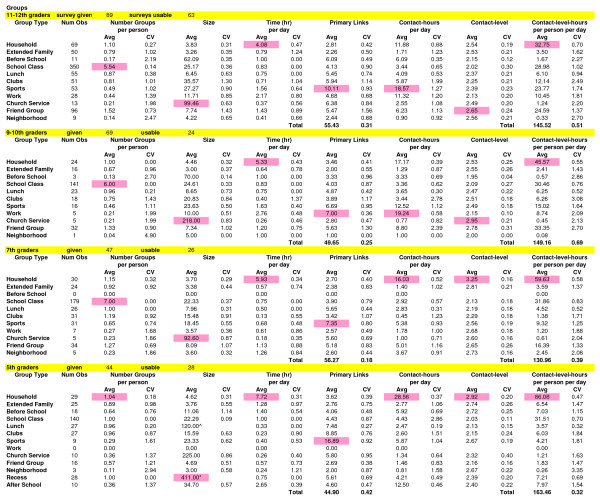
**Summary Statistics for Groups**. The highest average value (Avg) for each grade and group is shaded in pink. CV is the coefficient of variation. The number of observations (Obs) for groups is the total number of each kind of group across all people for each grade. Per-person values were calculated for the average student. Per-day values were formed for an average day that incorporated both week days and weekends. All terms are defined in **Table 1**. * denotes number of students in the school; ^ denotes one third of the school.

**Figure 2:**
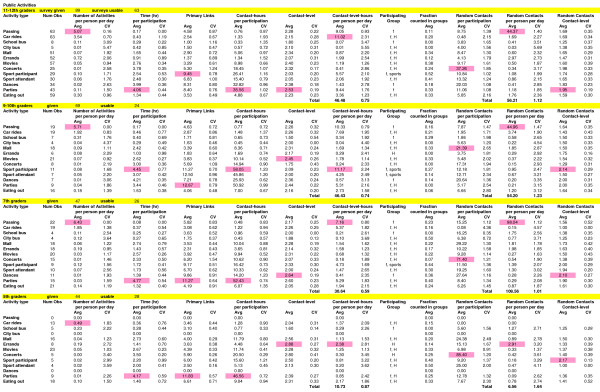
**Summary Statistics for Public Activities**. The highest average value (Avg) for each grade and public activity is shaded in pink. CV is the coefficient of variation. The number of observations (Obs) for public activities is the total number of people who had these activities. Per-person values were calculated for the average student. Per-day values were formed for an average day that incorporated both week days and weekends. The participating group is the group that the majority of students recorded doing a certain public activity with. H stands for household, f stands for friends, and sports for sports group. All terms are defined in **Table 1**.

For each student grade (or grade range), statistics are summarized for groups (Figure 1) and for public activities (Figure 2). We focus in our presentation below on contact-hours (quantity measure), contact-level (quality measure), and their combination into contact-level-hours (quantity-quality measure). We then address random contacts by public activity and age assortativity (the tendency for like age to assort with like) both for primary links in groups and for random contacts. Finally we consider individuals and variability (or heterogeneity) within the student population, their groups and their public activities.

### Contact-hours

Contact-hours show critical groups or public activities on a per-day basis for groups or a per-participation basis for public activities (if a person participates, these are the contact-hours they would have doing the particular activity once). Averaged across all grades, household groups have the highest contact-hours per-day overall but they decrease in magnitude from 5^th ^graders (29) to 11–12^th ^graders (12). With higher grades, sports, the second highest contact-hour group overall, increases with grade to become the highest contact-hour group for 11–12^th ^graders. Contact-hours in work per-day increase with grade and are largest for 9–10^th ^graders (primarily because 3 out of the 5 students who work in 9–10^th ^grade spend significant time in large family business work groups). Contact-hours in friend groups also increase with grade up to 9–10^th ^grade and then decrease slightly in 11–12^th ^grade. In public activities, contact-hours vary widely from under 1 (passing and school/city bus) to over 30 (dances, parties, or sport event participant/attendant). We note that the high contact-hours in certain public activities, while much higher than for any of those for particular groups, correspond to individual occurrences (events), many of which are infrequent.

### Contact-level

Contact-level indicates the quality or intensity of the social contact, the higher the level, the higher the potential of transmitting influenza. Contact-level averaged across all grades varies from near and below 2 for the school/city bus activities to above 2.5 for the household, extended family, church, friends, and sports groups (in decreasing order). No public activities are above an average level of 2.5 when averaged across grades. For individual grades, several additional groups have instances above a level of 2.5 including clubs (9–10^th ^grade), work (7^th ^grade, all babysitting), neighborhood (5^th ^and 7^th ^grade), and before school care (5^th ^grade), as well as have several public activities including parties (7^th ^and 11–12^th ^grades), dances (7^th ^grade), mall (5^th ^grade), errands (5^th ^grade), concerts (5^th ^grade), and participation in sport events (5^th ^grade). Contact-level averaged across all grades is slightly higher averaged across groups (2.4) than across public activities (2.2) but well within the SD of each other. Contact-level for random contacts is presented in its section below.

### Contact-level-hours

**Figure 3:**
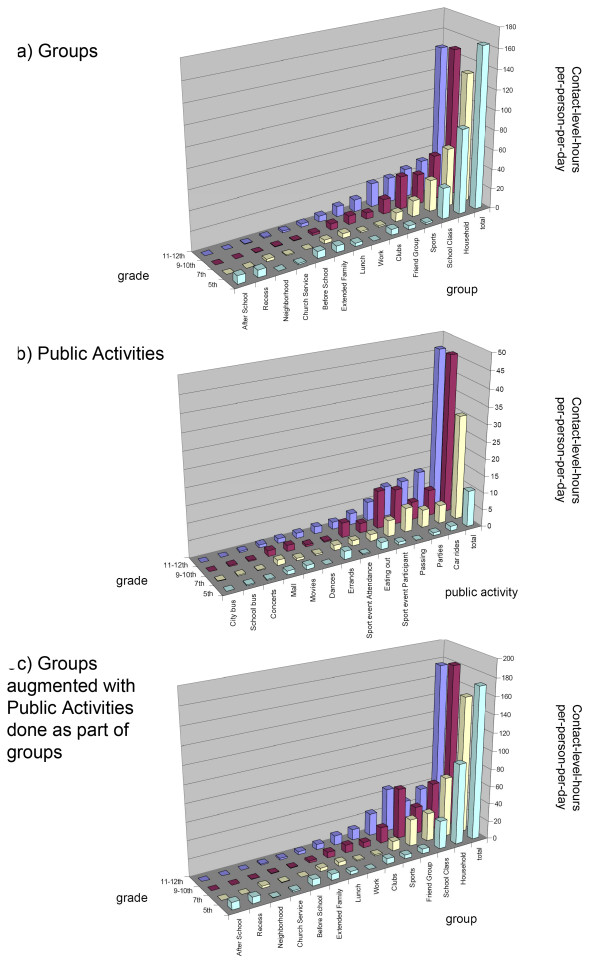
**Contact-level-hours by Grade and Group/Public Activity**. Contact-level-hours per-person-per-day is shown by grade and group/public activity with smaller values to the left and totals at the far right. Figure 3a) illustrates groups, 3b) illustrates public activities, and 3c) illustrates groups augmented with public activities, based on the participating group (for example, if students recorded going to dances with friends and these were not already included in time spent with friends, the contact-level-hours per-person-per-day in dances was added to the friend group). Per-person values were calculated for the average student. Per-day values were formed for an average day that incorporated both week days and weekends.

Contact-level-hours in each group or public activity is our best estimate of its potential for influenza transmission. Combining contact-level-hours with the number of groups or public activities a person has per-day yields a per-person-per-day value and allows groups and public activities to be appropriately compared and combined. These values have been plotted in Figure 3a and 3b to show the average contact-level-hours by grade, group and public activity, as well as their totals across groups or public activities.

In Figure 3a, totals for groups are highest in 5^th ^grade (163), dip in 7^th ^grade (131) and then are roughly the same in 9–10^th ^and 11–12^th ^grade (145–149). Households are most important in 5^th ^grade making up 53% of the total group contact-level-hours. The household group loses some of its importance to friend and sport groups as grade increases. School classes are nearly identical across all grades at approximately 30 contact-level-hours (19–24% of the total). The combination of household, school classes, friends and sports make up from 76 to 90% of the total contact-level-hours across grades. Contact-level-hours within the rest of the groups are small.

In general, contact-level-hours in public activities are much smaller than in groups. In Figure 3b, total contact-level-hours for public activities increase dramatically from elementary to middle to high-school, while within high school (9–12^th ^grades), they are similar. Contact-level-hours are largest within car rides, parties, school passing periods and sport event participation. Values for car rides and parties increase with grade in high school while those for passing and sport event participation decrease. In all remaining public activities, contact-level-hours are small. Interestingly, errands are the highest contact-level activity for 5^th ^graders (and 5^th ^grade errands are highest across errands for all grades); this reflects elementary school children accompanying their parents on household errands.

Nearly all students did public activities as part of either a household, friend or sport group. In the activity portion of the survey, we had asked students whether the time they recorded in each public activity had already been accounted for in their previously recorded group time. Most said that the time had not already been counted (see Figure 2). While adjustment of the statistics of individual groups for this response cannot be done precisely, we can make use of their answer to augment contact-level-hours in groups with those in public activities done as part of these groups and avoid double counting. Figure 3c shows all contact-level-hours across the student population augmented in this way. Comparison with Figure 3a shows the same essential trends with grade, the greatest increases in values are within the friend group for 7^th ^grade and above. The sum of group and public activity contact-level-hours per-person-per-day is similar for 5^th ^(172), 9–10^th ^(182) and 11–12^th ^(177) graders, with the dip in 7^th ^(154) grade still present.

### Random contacts

The average number of random contacts that occur outside of the primary links within groups when engaged in a public activity varies widely from highs at concerts (37–85) to lows in car rides (2 and below). Average contact-levels are uniformly low (near 1 for car rides up to 2.2 for participation in a sport event) and are lower than with primary links when in the public activity (see Figure 2). Random contacts may also occur in the work environment as people in service jobs interact with customers. Two out of five 9–10^th ^graders and five out of 28 11–12^th ^graders who worked came in contact with random customers (ranging from 60–200 per week) but these distribute to less than 2 random contacts per-person-per-day for each grade. Random contacts per-person-per-day in middle and high-school passing periods far exceed all other public activities at 98 for 7^th ^graders and 44–45 for 9–12^th ^graders. The fact that 7^th ^graders have roughly double the number of random contacts within passing periods is unexplained and could be due to differences in hallways, sequencing of classes within the school, density of the population, or simply that 7^th ^graders tend to stand and walk closer to others (bunch) than high-school teenagers.

### Age assortativity in groups and random contacts

**Figure 4:**
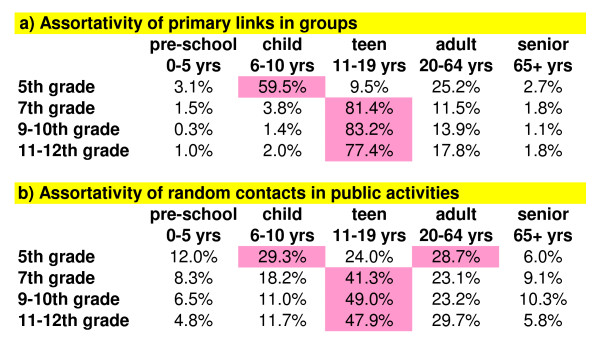
**Age Assortativity**. The percent of contacts between students in the surveyed grades and various age classes are shown for 4a) group primary links and 4b) random contacts. The highest percent of contacts are highlighted in pink. Assortativity is the tendency of a particular age class to assort with another.

Across all groups, the percentage of primary links between each grade and age class (preschool 0–5, child 6–10, teen 11–19, adult 20–64, senior 65+) are shown in Figure 4a. 5^th ^graders are children while 7^th ^through 12^th ^graders are teenagers. The vast majority of primary links are with those of the same age, as high as 77 to 83% for high and middle-school teens. While elementary-school children have 60% within age class, 25% are with adults primarily due to greater association with them in groups outside the household. The ages of randomly contacted people range from preschoolers to seniors and their fractions across all public activities are shown in Figure 4b. As with primary links, age class assortativity is also present in random contacts but to a lesser degree (all below 50% within age class).

### Individual students, variability and heterogeneity

**Figure 5:**
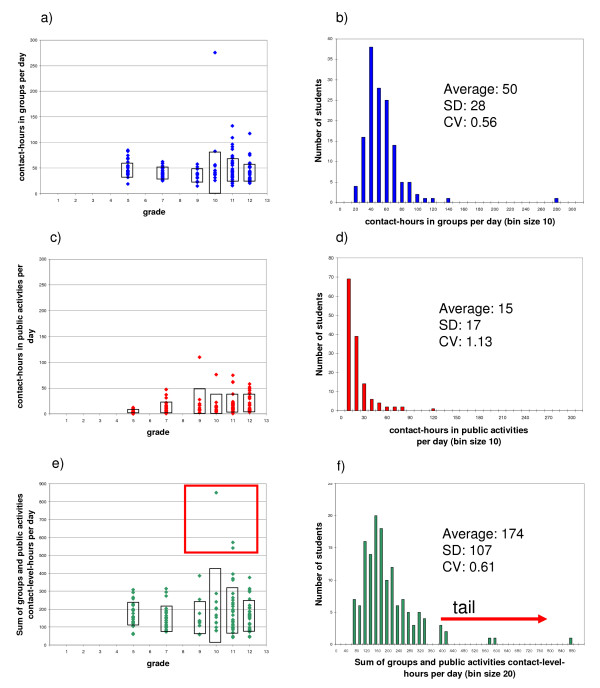
**Individuals**. We show for each grade the 5a) contact-hours in groups per-day, 5c) contact-hours in public activities per-day and 5e) the contact-level-hours per-day added across both groups and public activities without double counting. In each figure a point is plotted for an individual and a box denotes plus and minus one SD centered on the mean value. The red box in 5e) calls attention to the three data points as possible "super-spreaders" within the population surveyed. 5b), 5d), and 5f) show histograms that combine all students in all grades. Per-day values were formed for an average day that incorporated both week days and weekends.

For each individual student, the total number of groups they belong to, their contact-hours-per-day, average contact-level and contact-level-hours for both their groups and public activities were evaluated. In addition, the total contact-level-hours across both their groups and public activities were summed (adjusted to remove double counting as in Figure 3c). Across all analyses, we found no significant difference between genders. Distributions for the number of groups and level of contact in both groups and public activities are fairly narrow. However, for contact-hours and contact-level-hours per day, we find students to be highly heterogeneous. To demonstrate this heterogeneity, we show for each grade the contact-hours in groups per day in Figure 5a, the contact-hours in public activities per day in Figure 5c, and the total contact-level-hours per day in Figure 5e. In each figure a point is plotted for each individual and a box denotes plus and minus one standard deviation about the mean. Figures 5b, 5d, and 5f show histograms for Figures 5a, 5c, and 5e, respectively, that combine all students in all grades.

Across all grades, minimum contact-hours per-day are above 20 for groups while for public activities, there are always people with near zero contact hours. Histograms across all grades for both public activities and groups are skewed to lower values (the left in Figures 5b and 5d) with long tails to higher values (the right in Figure 5b and 5d). Total contact-level-hours per-day shows the potential for influenza transmission of individuals (Figures 5e and 5f). The shape of this distribution is skewed as are its components in Figures 5b and 5d. Of particular interest is the small number of individuals with very high contact-level-hours, the highest of which is 850, more than 6 standard deviations from the mean value of 174. There are 3 individuals above 500 (see red box in Figure 5e). Examining the survey questionnaires from these specific individuals, their high values come from a large number of primary links in all of their groups but especially friends, sports (wrestling in particular), clubs (particularly dance and drama) and work groups. These people could potentially serve as "super-spreaders" who would be more prone to both catch and spread influenza.

The heterogeneity seen across individuals is also reflected within the distributions for groups and public activities. In context of variability, the coefficient of variation (CV) is a measure of the width of the distribution rather than an indicator of error in the measurement. Across all groups and public activities, the CV for contact-level-hours per-person-per-day ranges from near 0.5 to above 3 (see Figures 1 and 2). Households, school classes, lunch, recess and passing periods have CVs of 1 or below while before school, clubs, church service, neighborhood, friends and many activities are 2 and above. Histograms of contact-level-hours for each of these groups/public activities with large CVs are highly skewed such as seen in Figure 5.

## Discussion

We have characterized a group-centric social contact network for school age children and teenagers that contains a student's primary links (within 3 ft and at least several minutes in length), the length of time that these links are active over the course of a typical day, and the typical level at which contacts occur. We have also characterized the public activities done as a part of these groups and the number, level, and setting of resulting random contacts. These data can be combined to assess the potential of influenza transmission within groups and public activities. Below we focus our discussion of these data in context of pandemic influenza to consider 1) critical features, 2) school closure and social distancing measures, and 3) limitations and extensions.

### Critical features

Both critical groups and public activities can be identified for the spread of pandemic influenza. Households and school classes are the two most critical groups with sports and friends joining them in higher grades. Public activities rise in importance from small for elementary-school children to nearly a third of that in groups for high-school upperclassmen. Car rides, parties, school passing periods, and participation in sport events are dominant public activities in the higher grades. While car rides and school passing periods occur often, parties, sport event participation, and dances are much less frequent but have both high contact-hours and high contact-levels when they occur and should be avoided during a time of pandemic. If such events were to occur during the pandemic's early stage, they might significantly hasten the spread of the disease within a community.

Outside of the households and structured groups within schools (such as classes), groups and public activities are highly heterogeneous. For example, contact sports such as wrestling have a much higher number of contact-level-hours than tennis. Individuals belong to many different groups yielding large variability in the contact-level-hours on a per-person-per-day basis in each group (for the average person) and from individual to individual across all groups. Present in the data are a small number of individual students with very high contact-level-hours that would likely be super-spreaders prone to both catch and spread the disease. This feature of the social contact network and the resulting skewed distribution is a common result in social networks and reflects the wide heterogeneity of people's behavior [16]. Similar skewed distributions have also recently been measured for the conversational contacts of school children ages 6–13 years [14]. During a time of pandemic, changing the behavior of people with high contact-level-hours may be important, especially for the super-spreaders themselves, as they are at high risk of becoming ill.

Interestingly, the number of people contacted at random during a middle or high-school (but not an elementary school) student's day rivals or surpasses the number of primary links within a student's social contact network (Figures 1 and 2). In terms of the number of different people within one's social contact network, the discrepancy is further magnified because primary links are sometimes duplicated across groups (for instance, some primary links within a friend group may be repeated in a sport group). The vast majority of these random contacts occur during passing periods in school which far exceeds those in malls or encountered within the work week of students in service oriented jobs. But random contacts are quick, of low contact-level, and thus yield negligible contact-level-hours per-person-per-day. We can estimate conservatively the possible contact-level-hours with random contacts by taking each contact of 1 minute duration and of level 2 yielding 2 per-person-per-day for 9–12^th ^graders, 4 for 7^th ^graders, and less than 1 for 5^th ^graders. On the other hand, one infectious person coughing throughout a single passing period during school has the potential to infect 8–15 other students (and there are up to 6 passing periods per day).

There is very strong assortativity by age for primary links within groups, and to a lesser extent for random contacts encountered within public activities. Thus, the vast majority of contacts for school aged children and teenagers are with their peers. All our values for assortativity are higher than found in previous studies for college students [17], the general population (and specifically children and teenagers) [18], or for grade school children [14]. The other studies of comparable age groups as ours [14,18], counted conversations between different people over a given length of time. Our survey included being near others (within 3 ft) without conversation (such as often occurs within classrooms) and this will increase the number of like with like contacts we record, especially within schools. Also, as mentioned above, primary links may have some overlap between different groups, each of which would be counted separately in our measure of assortativity. High assortativity suggests that influenza will spread quickly through individual age classes. The very high assortativity of high school students combined with their high number of contact-level-hours, above 75% of which are outside of the home, suggests that teenagers in high-school may form the local transmission backbone of the next pandemic.

### School closure and social distancing measures

School closure and social distancing has become an integral part of the USA's national strategy for community containment of pandemic influenza [22,23]. Can such strategies, aimed at critical groups and age classes, successfully reduce transmission and locally thwart a pandemic? While we cannot answer this question entirely without a full characterization of a community's social contact network, we can estimate the impact of closing schools and keeping children and teenagers home on the potential of influenza transmission within these age classes.

**Figure 6:**
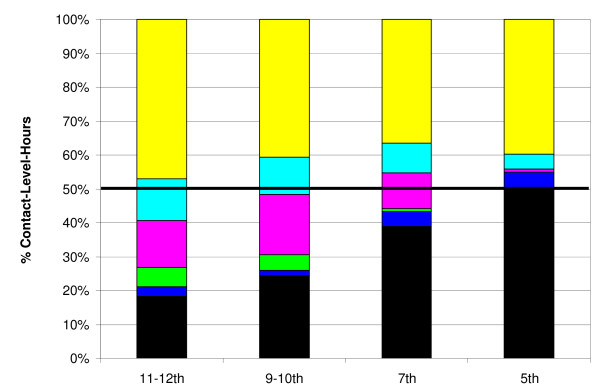
**School Closure and Social Distancing Measures**. Data are binned into the settings of school (yellow), non-school public activities (light blue), friends (magenta), work (green), neighborhood (blue) and household (black) expressed as a percentage of the total for each grade. For school (yellow) we have combined before school classes, classes, lunch, passing periods, and school bus rides and also all clubs, dances, sports, sport participant and attendance as all of these groups and public activities are school related. Neighborhood (blue) includes the neighborhood group as well as church and extended family. The black line represents a reduction to 50%.

While students, groups and public activities were highly heterogeneous, we use the average contact-level-hours per-person-per-day binned across a set of group types as our best estimate of the influenza transmission potential in these settings. Figure 6 shows our data binned into the settings of school, non-school public activities, friends, work, neighborhood and household expressed as a percentage of the total for the grade. For school (yellow) we have combined before school classes, classes, lunch, passing periods, and school bus rides and also all clubs, dances, sports, sport participant and attendance as all of these groups and public activities are school related. Neighborhood (blue) includes the neighborhood group as well as church and extended family. We see that contact-level-hours per-person-per-day can be greatly reduced by closing schools provided, of course, that students do not increase interactions in their other groups or public activities. Random contacts are also almost entirely eliminated through school closure (Figure 2).

If we were to close the schools and additionally keep all the children and teenagers home so that only the black in Figure 6 remained, the influenza transfer potential would be reduced to approximately 50% for 5^th ^graders, down to 18% for 11^th^-12^th ^graders, and all random contacts are entirely removed. While contact-level-hours within the household would necessarily increase for students kept there, the connection across these age classes within the community would be broken. In a household, even with many contact hours, the number of secondary cases remains limited. The epidemic must then be transmitted within the community by adults (assuming younger children are likewise kept home and the elderly do not play a significant role in transmission). If children and teenagers form the local transmission backbone of the pandemic and not adults, simulations have shown that closing schools and keeping students at home would be quite effective at quelling a pandemic's local spread [8,9]. Recent analysis of public health interventions applied during the 1918 influenza pandemic also have shown that closing schools in combination with other community containment measures were indeed effective [24-26].

### Limitations and Extensions

While others have found that observations of age specific risk of infection are well correlated with age-based self-reported social contact fractions [18], additional studies to consider bias in self-reported social contact networks have long been called for and continue to be needed [27]. Such studies would require accurate contact diaries to be completed and correlated with the same individual's questionnaire response. Nevertheless, the trends in the data reported here across age, groups and public activities are all quite reasonable and, while we do not know of studies of comparable detail, they fit with the "common knowledge" of those who live and grow up in suburban communities in the United States.

In the combined contact-hours (quantity) and contact-level (quality) measure of contact-level-hours, we have used a simple product that incorporates the expected trend in the potential for influenza transmission. Experimentally based mechanistic research, however, is required to improve this first order estimate. Depending on the disease, the combination of contact-hours and contact-level could and should be done differently, for example neglecting the level 1's if a disease can not be passed with a level 1 contact, or neglecting level 2 and below if physical contact is required. In this way, social contact networks characterized as we have in this paper could be used in context of other diseases that spread by human to human contact.

To facilitate and improve data gathering, a web-based interactive survey such as recently used by others [28] could be designed that first introduced influenza, pandemics, and social contacts and then guided individual students to complete the survey questionnaire. Entry of data directly and checks on answers that are out of bounds followed by additional queries of the student for resolution and non-specific prompting would remove many of the problems that restricted the number of usable survey questionnaires in the current study. Through such an approach, sample size could be greatly increased to yield a more complete characterization of the student community in a particular school system. If implemented as part of the curriculum, longitudinal studies could also be accomplished.

Albuquerque is a medium sized (population 500,000), suburban city. It has several high schools, Albuquerque High and its feeder middle and elementary schools are diverse in ethnic and socio-economic background. Extending this survey to other high schools and school systems may show some differences. The survey approach can also be modified and extended to pre-school centers, workplaces, and senior centers to capture the social contact networks of younger children, adults and the elderly. Such characterization would yield the full social contact network for a community and allow the tailored design of pandemic influenza containment strategies using approaches such as Glass et al. [8,9]. Applying the approach to other cities, states and nations would allow community specific characterization and cross-community comparisons to be drawn. Significant differences are expected across age classes, between rural and urban or developed and developing countries, and across cultures.

## Conclusion

We developed and applied an approach to characterize social contact networks for the spread of influenza within school aged children and teenagers. The network was conceptualized as of a number of groups to which people belong (such as households, school classes, clubs). Within groups, primary links which are both close (less than 3 ft) and extended in time (at least several minutes) were identified and characterized to yield the quality and quantity of contact (contact-level-hours) within each group. Public activities done as part of a group venturing into the community where random contacts occur (such as friends going to a movie) also were captured. This approach was applied in Albuquerque, New Mexico, a mid sized suburban city in the southwest of the United States.

The student population, as well as the groups to which they belong, were found to be highly heterogeneous. However, general patterns are clearly evident and allow identification of critical groups and public activities for the potential transmission of influenza. The importance of the household, school classes, friends, and sport groups are paramount with household decreasing and friends and sports increasing in importance with age. Public activities rise in importance from small for elementary-school children to nearly a third of that in groups for high-school upperclassmen. Public activities such as parties, sport event participation, and dances are infrequent but have both high contact-hours and high contact-levels when they occur and should be avoided during a time of pandemic. Random contacts during public activities in a typical day rival or surpass the number of primary links for all but elementary school children. However, these random contacts, largely in passing periods during school, amount to less than 3% of the total contact-level-hours per-person-per-day. Also present are a small number of individual students with very high contact-level-hours that would likely be "super-spreaders" prone to both catch and spread the disease.

Importantly, students are found to be highly assortative and tend to interact mainly within age class (like with like contacts). High assortativity suggests that influenza will spread quickly through individual age classes and schools. The very high assortativity of high school students combined with their high number of contact-level-hours (above 75% of which are outside of the home) suggests that teenagers in high-school may form the local transmission backbone of the next pandemic.

In light of our data, analysis of the community containment strategy of closing schools and keeping children and teenagers at home during a time of pandemic shows that such a strategy would be quite effective at removing the potential of influenza transmission within school aged children and teenagers. If they do form the local transmission backbone of the pandemic and transmission within the adult population alone cannot support its local spread, such a strategy may also be able to stop pandemic influenza within individual communities much as thinning a forest removes the potential for fires to grow, no matter from where they initiate. However, full evaluation of such strategies requires computational simulation that combines the social contact network of school aged children and teenagers with those of the remaining age classes within a community (younger children, adults, and the elderly). Continued effort along these lines can remove one of the largest uncertainties in the design of effective community containment for pandemic influenza – the social contact network.

## Competing interests

The author(s) declare that they have no competing interests.

## Authors' contributions

LMG designed, conducted and analyzed the results of this study, drafted the manuscript, and helped revise the manuscript based on the comments of the reviewers.

RJG advised and oversaw the study, helped draft the final manuscript, and responded to the comments of the reviewers.

Both LMG and RJG have read and approved the final manuscript.

## Pre-publication history

The pre-publication history for this paper can be accessed here:



## Supplementary Material

Additional file 1 **Supplementary information**. A. Survey overview prepared for the Albuquerque Public School Review and Clearance Committee; B. Description of social contact network conceptualization; C. Approach used for administration of survey questionnaire in classrooms; D. Survey questionnaire (blank); and E. Survey questionnaire (filled in example). Click here for file
