# Supplementary material for: Social contact networks for the spread of pandemic influenza in children and teenagers
Source: BMC Public Health. 2008 Feb 14;8:61. doi: 10.1186/1471-2458-8-61 (PMC2277389; doi:10.1186/1471-2458-8-61)
Supplement: Additional file 1 — Supplementary information. A. Survey overview prepared for the Albuquerque Public School Review and Clearance Committee; B. Description of social contact network conceptualization; C. Approach used for administration of survey questionnaire in classrooms; D. Survey questionnaire (blank); and E. Survey questionnaire (filled in example). [file 1471-2458-8-61-S1.pdf]

Social contact networks for the spread of pandemic influenza in children and teenagers

Laura M. Glass[1], Robert J. Glass*[2,3]

[1]Albuquerque Public Schools, Albuquerque, New Mexico, USA
[2]National Infrastructure Simulation and Analysis Center (NISAC)** Albuquerque, New Mexico, USA
[3]Sandia National Laboratories (SNL)*** Albuquerque, New Mexico, USA

* RJG is the corresponding author.
**NISAC is a program of the Department of Homeland Security's Infrastructure Protection/Risk Management Division and comprised of a core partnership of Sandia National Laboratories (SNL) and Los Alamos National Laboratory (LANL).
*** SNL is a multiprogram laboratory operated by Sandia Corporation, a Lockheed Martin Company for the United States Department of Energy's National Nuclear Security Administration under contract DE-AC04-94AL85000.

Email addresses:
LMG: arual721@comcast.net
RJG: rjglass@sandia.gov

Supplementary information

Table of Contents:
A. Survey overview prepared for the Albuquerque Public School Review and Clearance Committee …………………………………………………………...2
B. Description of social contact network conceptualization ……………………………..3
C. Approach used for administration of survey questionnaire in classrooms……….......4
D. Survey questionnaire (blank)……………………………………………………....14
E. Survey questionnaire (filled in example)…………………………………………..24

A. Survey overview prepared for the Albuquerque Public School Review and Clearance Committee

Characterizing social contact networks for the spread of infectious diseases

Laura M. Glass, Albuquerque Public High School

Introduction:

The spread of infectious diseases is important in today's highly connected world. Infectious diseases spread on social contact networks. Computer models can use these networks and the specifics of a disease to simulate its spread and help us figure out how to best stop or contain it. I have done this for pandemic influenza using a social contact network built from my own and other's experience. Can I do better? Can I characterize these networks? And will using characterized networks lead to better approaches to stop the spread of pandemic influenza?

Purpose of study:

1. Develop and apply an approach to characterize social contact networks
2. Analyze results to understand basic features of social contact networks
3. Analyze results to build a contact network appropriate for influenza within the Loki-Infect model (Glass et al. 2005, 2006)
4. Use the Loki-Infect model to find the best ways to stop pandemic influenza and compare to previous results

Characterization approach:

1. Classroom survey (gives expected typical social contact network data, it is based on a person's reflection and estimation of what they do)
2. Analysis of data and general description of measured contact networks
3. Distillation of data for use in Loki-Infect (like Table 1 in Glass et al. (2006))

Classroom survey plan:

In each class that I conduct my survey I will first cover the Introduction, Purpose of Study and Characterization Approach outlined above. I will then go over the survey quickly. Each individual will then fill the survey questionnaire out section by section with discussion/questions as we go along. This should help everyone construct the data I require on the groups that they belong to, their makeup, how often they meet, and the level of contact in each group setting.

References:

Glass RJ, Glass LM, Beyeler WE: **Local Mitigation Strategies for Pandemic Influenza**. In.: Prepared for the Department of Homeland Security under the National Infrastructure Simulation and Analysis Center; Report no. SAND2005–7955J. 2005.

Glass RJ, Glass LM, Beyeler WE, Min HJ: **Design of Targeted Social Distancing Strategies for Pandemic Influenza**. *Emerging Infectious Diseases* 2006.

B. Description of social contact network conceptualization

The following is quoted from:

Glass RJ, Glass LM, Beyeler WE, Min HJ: **Design of targeted social distancing strategies for pandemic influenza**. *Emerging Infectious Diseases* 2006.

Methods

The design process first creates an explicit social contact network in which persons are linked to others in a community. Spread of influenza within the network is then simulated by imposing behavioral rules for persons, their links, and the disease. These rules are modified to implement targeted mitigation strategies within the community, the effectiveness of which is evaluated (*5*).

Contact network

A network is created by specifying groups of given sizes (or range of sizes) within which persons of specified ages interact (e.g., school classes, households, clubs). The average number of links per person within the group is also specified because cliques form or are imposed (e.g., seating in a classroom). This number is used to construct a within-group network that can take various forms. We used fully connected, random, or ring networks for each group. Random networks are formed by randomly choosing 2 persons within the group and linking them. This process is repeated until the number of links within the group yields the specified average (each person will have a different number of links). The ring is formed by first placing persons next to neighbors and linking them to form a complete circle. Additional links are then made to next nearest neighbors symmetrically around the ring. Finally, links within a group are given an average frequency of contact per day. With this approach, a network can be built from the experience of community members to exhibit the clustered yet small-world characteristics (*6*) and overlapping quality of a structured community (*7*,*8*).

References:

5. Glass RJ, Glass LM, Beyeler WE. Local mitigation strategies for pandemic influenza: prepared for the Department of Homeland Security under the National Infrastructure Simulation and Analysis Center. Report no. SAND2005–7955J. 2005.
6. Watts DJ, Strogatz SH. Collective dynamics of ‘small-world’ networks. Nature. 1998;393:440–2.
7. Palla G, Deranyi I, Farkas I, Vicsek T. Uncovering the overlapping community structure of complex networks in nature and society. Nature. 2005;435:814–8.
8. Newman ME, Park J. Why social networks are different from other types of networks. Phys Rev E Stat Nonlin Soft Matter Phys. 2003;68:036122.

C. Approach used for administration of survey questionnaire in classrooms

Preliminary:

- Estimated time: 1-2 hours.
- Signed consent forms may be required depending on specific school requirements.
- Having the teacher participate in administering the survey greatly enhances the quality of the filled in questionnaires and the student's experience.

Overheads prepared ahead of time:

- Title of study, your name, contact information, approval if required.
- Simple bulleted purpose.
- Me and my links social network.
- Companion survey for student reference (I fill in an example survey as they fill in their own).

Introduction:

- *Show **Figure 1** Introduction slide.*
- The spread of infectious diseases is very important in our highly connected world.
- With modern technological advances in travel (air, bus, car, etc.), diseases have the potential of becoming pandemics more now than anytime in our history.

Introduction

- The spread of infectious diseases is important in today's highly connected world.
- Infectious diseases spread on social contact networks. Computer models can use these networks and the specifics of a disease to simulate its spread and help us figure out how to best stop or contain it. I have done this for pandemic influenza using a social contact network built from my own and other's experience.
- Can I do better? Can I characterize these networks? And will using characterized networks lead to better approaches to stop the spread of pandemic influenza?

Figure SI1: This is the introduction overhead that I used for this study.

- A *Pandemic* is simply the worldwide outbreak of a disease.
    - Pandemics can be extremely destructive, the 1918 Spanish flu pandemic killed more than 50 million people world wide.
    - For example, has anyone heard of the “bird flu?” There is a worldwide scare that this infectious disease (H5N1 virus) will one day soon become a pandemic, in which case we would all want to be prepared.
    - Discuss pandemics with the students, this gets them excited, especially when you bring up that understanding social networks could be used for social distancing strategies and that that could mean school closure!
- Infectious diseases such as the flu spread along social contact networks.
- A social contact network is basically the groups that a person belongs to in which they interact with others where an infectious disease such as the flu might be passed.
- *Show **Figure 2** “Me and My Links” slide.*
- Here is an example of my social contact network. There I am in the middle and as a big red circle in each of the surrounding groups. I am a part of several groups that I meet with on a regular basis in which I interact with others: Family, Extended family, Household, Friends, Sports, Work, 6-School classes, and the MESA club.

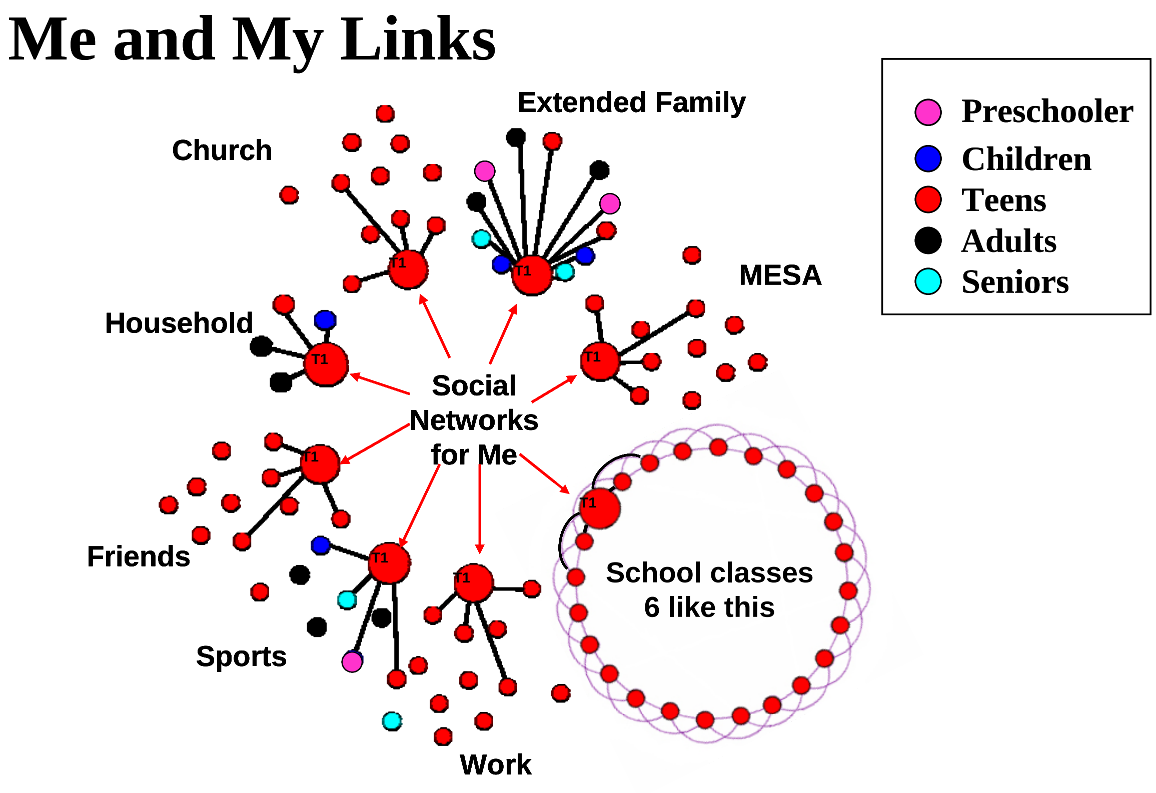

Figure SI2: When introducing the concept of a social contact network, I found a overhead slide like this to be very helpful and informative for the students.

- As shown in the slide, people are circles and of different *ages* (color), groups are of different *sizes* and are composed of people. In each group, I don't contact everyone in a way that would spread the flu. To catch the flu you need to be at least within 3 ft of a person who has it and is coughing and sneezing. The virus is in the saliva and mucus in your nose, eyes and mouth. So by touching a sick person you can also get the flu because they have their sneezes all over themselves, also the surfaces around them. The chance of passing the flu goes up if you are talking with the person face to face, or talking and touching the person, or kissing the person. We call these the *level* of the contact. Those people that I contact in any of these ways in each group, like the people I sit next to in a school class, are my *primary links* in the group for the transfer of the flu. And the chance of being coughed or sneezed on will be related to the *time* I spend in each group. Go through each group on the slide and describe them in terms of *size, primary links, time, and contact level* to make these ideas sink in with the students. This is how we are going to characterize their social contact networks (you may want to put this overhead up again from time to time as they fill in the questionnaire).
- Besides these groups, we do a lot of things out in the community like going to movies or the mall. When we do these *Public Activities* we usually do them with Friends or Family or one of the other groups we have shown in the slide. And when we do these *Public Activities*, we are likely to have other contacts with people in the community where we might be within 3 ft or talking or touching. These we are going to call *Random contacts* and we will be characterizing them too.
- Everyone has their own unique social contact network.
- Why is it important to characterize and understand these social contact networks?
- If we were faced with a pandemic soon, in this country and globally, there is no guarantee that there would be enough vaccine or anti viral medication to combat its spread. In addition, there is a big possibility that the vaccine and antiviral drugs that we have currently will not be very effective against a pandemic strain of influenza.
- In this case, we would need to turn to alternative strategies to avoid having the pandemic run wild through our community killing many.
- Computer models, which incorporate the concept of an abstract social network into their simulations (just like the one in the figure), have shown that social distancing strategies may be effective to limit the spread and destruction of a pandemic.
- Social distancing just means that we disrupt the social contact network by separating people or entirely disbanding the group for the time of the pandemic, like closing schools.
- Social distancing strategies can be designed to target a specific age class or set of groups or public activities defined as important in the spread of disease, that is, cut the most critical connections in the social contact network and hinder the spread.
- For example, my simulation studies in the past two years have shown that even a simple measure such as school closure during time of a pandemic can be highly effective in curbing the destructive path of infectious disease. This would only work, however, if students stay home rather than spending more time mixing in malls or with friends, which would only spur on the spread of disease.
- Therefore, if we are able to understand the social contact networks of various age classes in a community, we can design possible social distancing strategies that target

those highly important groups to limit the spread of the disease without the use of vaccine or anti-viral drugs.
- This type of thinking has begun to shape the United States' planned response to a possible flu pandemic.

Administering the survey:

Initial Directions:
- It is important that the survey questionnaire be filled out in pencil so that students can correct mistakes neatly, making it easier to decipher later. Before passing anything out, ask all students to pull out a pencil.
- Be sure to come with many extra pencils, and ask students to raise their hands if they are in need of one.
- Pass out a survey questionnaire to each student and teacher (though we did not include teachers in our analysis, it would be interesting to capture the school environment from a teacher's point of view).
- As they get their survey questionnaire, ask students to quickly glance through it, so they see what is coming.
- When they receive the survey questionnaire, have students fill in their class at the top, but to not go on until you give them further instruction (otherwise, students make many mistakes).
- Make sure that you have an overhead survey questionnaire ready beforehand as an example for students to refer to. I found it helpful to fill in the survey questionnaire for myself as they filled in theirs as it made it easier to explain, and kept them from looking ahead and becoming confused.

Groups section: Pages 1-5

Pg 1, Household and Extended Family:
- After all students have received a survey questionnaire and have filled in their class, ask them to turn their attention to the first table labeled "Household Members".
- Explain that this first part of the survey questionnaire is to get information on their household, or family.
- Ask them to simply fill in the first row of the table titled "yourself", it is pretty self-explanatory (Initials, Age, Gender, Occupation or grade, shared room (yes/no)). Again stress that they do not go on alone.
- Throughout the process, it may be helpful to ask students to show a sign that they are ready to continue, such as putting their pencils down on their desks.
- Have students fill in the first table for each member of their household. If they are a part of more than one household, ask them to leave a space between the two, and record them both. Also, if they should need more room, have extra sheets available for them to fill in.
- Go through each column and explain the meaning and significance:
    - *Relationship* would be father, brother, mother, grandmother, etc.
    - *Initials* of that person.
    - *Age* of that person.

    - *Gender* of that person.
    - *Occupation* (primary) or grade if that person is a full-time student.
    - *Time* spent with that person on a daily basis (make sure that they understand to think about this as an average weekday, consider the amount of time that they are within 3ft of that person for greater than several minutes. Stress that they don't need to become frustrated when recording time, it is simply an AVERAGE value for an AVERAGE weekday for this section). You should talk through an example of how to do this, possibly using a student from the class (example: I interact with my sister before school for about 20 minutes, after school for about 1 hour, and before I go to bed for about 40 minutes, so I spend on average 2 hours with my sister each weekday).
    - *Contact level* with link (person). Explain to students that although a person may have many contacts with others throughout a day, some contacts are more important or dangerous than others with respect to the spread of disease. For example, just sitting next to a person in class may not be as important in the spread of disease as kissing. Explain the four levels, 1: close, within 3 feet, 2: close and talking, 3: close, talking and touching (nudging, hugging, etc.), 4: kissing.
    - *Shared room* in the household (bedroom).
- Ask students to put down their pencils when they are ready to continue.
- Explain the question "do you spend additional time with household members during the weekend? If so, how much?"
    - They can either write no, or yes. If they write yes, they must specify about how much extra time they spend with household members on the weekend as a whole, or, if they prefer, separating out Saturday and Sunday.

- Next, have students place their attention on the next table "Extended Family Members".
- Bring up the point now and every once in while as the students are filling in the survey questionnaire that, if on any part of the survey the student feels that he/she does not have a particular group, like a 5th period class, or an extended family that they see regularly, they MUST write in *None* in its place.
- Make sure that the students understand that if they simply leave something blank, you can not tell whether they don't have that particular group/public activity, or if they simply forgot and skipped it.
- Continue to fill in an example with them on the board or overhead, and let them know that the extended family portion is to be filled out exactly as the household section, except that they should record time spent with a person *on a weekly basis, rather than daily.*
- Point out that the extended family should not include friends or neighbors as those are separate groups that will be brought up later in the survey questionnaire.
- Ask that students record their extended family by household (aunt and uncle may be in one household, grandparents in another for example).
- Again, ask that the students do not simply move on when they are finished with the extended family section but that they put down their pencils and look up when they

are done, as the next page which assesses school classes is slightly different from the first.
- I found it helpful to let them know that after the next page, they should be able to go through the rest of the groups portion of the survey questionnaire on their own if they wish, which prevented the students who were quick from getting restless and impatient.

Pg 2: School Classes:
- Once all students have completed the first page and are ready to move on, ask them to turn to the next page with “Group Name” in the left-hand corner.
- State the different headings quickly, then show them the details by filling in a couple class examples for yourself, or for an example student on the overhead.
    - *Group name*. Simply the name of the group, for school classes these are your classes during the day. Make sure that you fill out the name of each class under the period number.
    - *Time* in group type per day (an average weekday). Classes are usually the same length of time.
    - *Size*. This is the average (or exact) size or size range of the group, just the number of people in a class. The size would include yourself.
    - *Age range*. The age range of the entire group, from youngest to oldest (include the teacher separately).
    - *Number of primary links* (people) in your group and *initials of primary links*. Include teacher or coach if applicable.
        - This concept is at first daunting and difficult for most students to grasp, so a small explanation and an example is essential.
        - Although you may have 35 people in a class, you may not actually have close interactions with all 35 of those people. For instance, in many classes you are often just sitting down, not mingling with every student in the class. To explain this, perhaps choose a student as an example. Ask that student how many people are within 3ft of him/her when they are sitting down, and whether he routinely has extended interactions with any other people in the class, and if so, how many. Perhaps the student has 3 people sitting around him/her, always interacts with the teacher and a couple of other students, so the number of primary links would be 6 out of the entire class.
        - Initials are recorded so that students are actually thinking through specific people that they interact with on a daily basis in a specific class, rather than making wild guesses. Also, if one wished, they could consider how many times a person will interact with the same person but in different groups within their social contact network. Mention to the students that if they come across a person who has the same initials as someone else, put a 2 next to it, so that I will know that it is a different person.
            - You may notice that there are two boxes for this category. The smaller, left-hand box is for the number of primary links and the larger, right-hand box is for the initials.

- *Relationship with primary links.* F: Family, f: friend, A: authority, a: acquaintance.
    - This column also serves to find patterns and to allow the students to carefully think through their contacts with specific people throughout the day. Make sure that the students know, through your example on the overhead, that they should put the letter of the relationship with a specific person in the corresponding place that the initials were in.
    - Explain that F is a family member, f a friend, A for authority is a person like a coach, or teacher, or boss, and a: for acquaintance is for a person that you know, but you wouldn't call a friend.
- *Contact Level with primary links*. 1: close, 2: talking, 3: touching, 4: kissing
    - Explain to them that this is the same as before and briefly outline the four levels again.
    - Emphasize that the contact level with a specific primary link must, similar to the relationships, be in the same corresponding place in the box.
- *Ages or age range for primary links.*
    - If they know the exact age for each of their primary links, ask students to place the age in the same corresponding place as the initials for that primary link. If they do not know the exact ages for all primary links, ask them to write down an age range for them.
- Have all students fill in this section of the survey questionnaire for all their classes, with yours as an example. Walk around the room to make sure that everyone is on the right track and answer any questions.
- Have students wait when they finish this page, there is one more explanation you should make before they are allowed to move on independently for the rest of the group section.

Pages 3-5, More Groups:

- When all students have completed the second page assessing classes, ask them to quickly glance through the remainder of the survey questionnaire before they begin, so that they do not end up including a group that is near the end, such as Neighborhood under an earlier group such as School Clubs.
- Quickly state that this part of the survey questionnaire will assess the kinds of groups that they belong to, settings and environments that they are regularly a part of, like School Sports, or Clubs.
- State each of the groups that is mentioned: School Clubs, Non School Clubs/Lessons etc., Sports, Work, Church Service, Friend groups, Neighborhood, and Other.
- The only thing that is different from the 2nd page that looked at School Classes and the rest of the survey questionnaire is that the *time should now be recorded on a per week basis, including the time you might spend in the group during the weekend.*
- Also, let students know that there are multiple spaces in the case that they have more than one group of a particular group type. And remind them that if at any point they come across a group that they do not have, School Clubs for example, they MUST record *None* in the space provided.

- **Special Notes for Particular Group Types:**
  - *Sports***:** include all that you participate in during the year and note which season in the year with the group name. For the time spent in the sport, include the weekend, meets, etc.
  - *Work***:** make sure that you fill in the name of the place that you work and the type of work that it is (receptionist, waitress, etc.). Fill in the number of coworkers under the group, and then the customers that you interact with below.
  - *Church***:** Be sure to record the name of the Church that you attend. For the group size, include all of the people that you think are in each (service, Sunday-school, etc.). The primary links will be the people that you go to church with, the people you sit with, and those people that you interact with in a church setting on a regular basis.
  - *Friend Groups***:** if you are a part of more than one group of Friends who do not interact with each other, put them as separate groups. These could be Friends from Sports and Friends from school for example. Time spent with a Friend group is only the time spent outside of school classes, lunch, etc. Make sure that you mention that students should not include time that they spend sitting next to a friend in class in the time spent in a Friend group as it is already counted there.
  - *Neighborhood***:** if there are people in your Neighborhood that are included in you friend groups, do not include them here.
- At this point, you can let the students loose on the survey questionnaire, make sure that you keep up with examples of a filled in survey questionnaire on the overhead, and walk around and make sure everybody is on track, answering any questions.
- Say several times that if there are groups or interactions that don't seem to be captured on this form, put them in under OTHER at the bottom.
- Make sure that you tell students that when they are finished with the groups part of the survey, that they do not go on to the Public Activities section without further instruction because it is much different.

Public activities section: Pages 6-7

- When administering the survey, as soon as a fair number of students reach the Public Activities section, ask that all students turn to the Public Activities section so that you will not have to explain it more than once, and so that students ready to move on, can.
- This section of the survey is designed to capture those activities that a person may do alone or in a group venturing out into the public community where many unplanned interactions may take place. Talk this over with the students to make sure that they understand this concept.
- As done with the groups section of the survey, first have students look at all of the public activities that have been called out, to get them thinking of how a public activity is classified, and how they differ from groups.

- Next, go through each of the separate columns quickly first, then take them through a couple examples more slowly, explaining the headings as you go.
    - *Activity*. These are given (Passing Periods in schools, Before-After school, Car rides, School bus, City bus, Mall, Errands, Movies in theatres, Concerts, Sport/game event Participant, Sport/game event Attendant, Dances, Parties, Eating out), you can again reiterate what constitutes a public activity as defined above.
    - *Number of times* per day, week, month, or year. As public activities may not be done regularly, students must think about an average day, week, month or year and record the number of times, or a range that they might do a particular public activity. Emphasize that students *must* record if they participate in the public activity per day, per week, per month, or per year. Just a number will not be sufficient in analysis.
    - *How much time* does this public activity take? (each time you do it). Again, this is an estimate, and can also be a range.
    - *Which groups* do you do this activity with? As stated in the definition of a public activity, they are activities that one may do alone or as *part of another group* venturing out into the public community. So, students should record the groups if any, that they have previously defined in part one of the survey, that they participate in a particular public activity with (going to the movies with a friend group, for example).
    - *How many group members*, or *primary links* do you have contacts with when you do this public activity (your primary links): this question is designed to separate random connections with unknown people that may occur in a public activity, from interactions with people that you know or are a part of your group.
    - *What is the range of level of contact*? for these group members (or primary links), same levels as before in the groups section of the survey questionnaire.
    - *Is this activity already included in the time you spend in other groups*? This question is designed to help avoid double counting, such as time going to the movies that has already been counted into a Friend group. This is a yes or no question.
    - *How many non-group members* do you have contacts with when you do this public activity? This question is designed to capture the random aspect to a student's social contact network, this may be a carefully estimated range.
    - *What is the range of level of contact* with these *non group members*? Again, this may be a range, and the levels are the same as before.
    - What is the *age range* of these *non group members?* Similarly, a carefully estimated range.
- Finally, the last question is aimed at checking that students did not overestimate or underestimate the amount of time that they spend in groups and public activities with others, a quick check. "Amount of time spent per day doing things on your own (homework, TV, reading, etc.)"

Finishing:

- Have the students go back over their survey questionnaire and check their work. This may involve them re-estimating some times, group sizes, levels, etc.
- When students have finished the survey questionnaire, collect them as they finish.
- Try to flip through the survey questionnaires as you receive them, to catch anything blatantly incorrect or incomplete in the survey questionnaires.
- As students hand in their survey-questionnaires, you may hand them an additional mini-questionnaire which queries their perceived compliance for social distancing targeted at their age class. While this is not a necessary aspect of the survey questionnaire and was not used in this study for subsequent analysis, it was interesting to hear the point of view of the students on this matter, and kept students busy that finished early.

Student feedback:

- If there is time, ask the students the following questions. Their answers may be used to improve the survey questionnaire and your method of delivery.
    - Which part(s) was/were most difficult to figure out?
    - Did you have any problems?

D. Survey questionnaire (blank)

Social Contact Survey Questionnaire:

Class: ________________________________

Household members:

Relationship	Initials	Age	Gender	Occupation or grade	Time spent with person on a daily basis (weekday)	Contact level with link (person) 1:close 2:talking 3:touching 4:kissing	Shared room? (yes/no)
Yourself					*Na*	*Na*	

Do you spend additional time with household members during the weekend? If so, how much?	

Extended Family members visited regularly (non household): group by their household

Relationship	Initials	Age	Gender	Occupation	Time spent with person on a weekly basis	Contact level with link (person) 1:close 2:talking 3:touching 4:kissing

Group Name	Time in group per day (weekday)	Size (number of people in group)	Age range in group	Number of primary links (people) in your group and initials of links, include teacher or coach if applicable	Relationship with primary links<br>F: Family<br>f: Friend<br>A: Authority<br>a: Acquaintance	Contact Level with primary links<br>1:close<br>2:talking<br>3:touching<br>4:kissing	Ages or age range for primary links
School							
Period 0							
Period 1							
Period 2							
Period 3							
Period 4							
Period 5							
Period 6							
Lunch							

Group Name	Time in group per week (include weekend)	Size (number of people in group)	Age range in group	Number of primary links (people) in your group and initials of links, include teacher or coach if applicable	Relationship with primary links<br>F: Family<br>f: Friend<br>A: Authority<br>a: Acquaintance	Contact Level with primary links<br>1:close<br>2:talking<br>3:touching<br>4:kissing	Ages or age range for primary links
School Clubs							
Non school Clubs/lessons/ etc.							

**Group Name**	**Time in group per week (include weekend)**	**Size (number of people in group)**	**Age range in group**	**Number of primary links (people) in your group and initials of links, include teacher or coach if applicable**	**Relationship with primary links F: Family f: Friend A: Authority a: Acquaintance**	**Contact Level with primary links 1:close 2:talking 3:touching 4:kissing**	**Ages or age range for primary links**
Sports							
Work							
	xxxxxxxx	xxxxxxxx	xxxxx	xxxxxxxxxxxxxxxxx	Number of customers	Contact level	Age Range
Church							
Service							

Group Name	Time in group per week (include weekend)	Size (number of people in group)	Age range in group	Number of primary links (people) in your group and initials of links, include teacher or coach if applicable	Relationship with primary links F: Family f: Friend A: Authority a: Acquaintance	Contact Level with primary links 1:close 2:talking 3:touching 4:kissing	Ages or age range for primary links
Friend groups							
Neighborhood							
Other							

**Public Activity**	**Number of times per day, week, month, or year**	**How much time does this public activity take? (each time you do it)**	**Which** *groups* **do you do this public activity with?**	**How many** *group members* **do you have contacts with when you do this activity? (your primary links)**	**What is the range of level of contact with** *group members* **(primary links)?**	**Is this public activity already included in the time you spend in other groups?**	**How many** *non group members* **do you have contacts with when you do this public activity (random contacts)?**	**What is the range of level of contact with these** *non group members* **(random contacts)?**	**What is the age range of these** *non group members* **(random contacts)?**
Passing Periods, before-after school									
Car rides									
School bus									
City bus									
Mall									
Errands									
Movies in theatres									

Public Activity	Number of times per day, week, month, or year	How much time does this public activity take? (each time you do it)	Which groups do you do this public activity with?	How many *group members* do you have contacts with when you do this activity? (your primary links)	What is the range of level of contact with *group members* (primary links)?	Is this public activity already included in the time you spend in other groups?	How many *non group members* do you have contacts with when you do this public activity (random contacts)?	What is the range of level of contact with these *non group members* (random contacts)?	What is the age range of these *non group members* (random contacts)?
Concerts									
Sport/game event participant									
Sport/game event attendance									
Dances									
Parties									
Eating out									

Amount of time spent per day doing things on your own (homework, TV, reading, etc.)	

Additional Questions: (if time allows)

1. When you are sick, do you tend to stay home, or go about your business as usual?

____

2. What about your parents/guardians?

____

3. If schools were closed due to threat of a major disease, and you were told to stay home without seeing your friends, going to movies or the mall, and only having physical contact within your household, how difficult would this be for you, would you obey?

____

4. If you were then informed that if you got the disease you had a 1 in 10 chance of dying, would you obey?

____

5. If you work, are people closer together in a work environment, or a school environment?

____

6. Do you have higher level contacts at school or work?

____

7. In which group or activity do you think you have the highest chance of passing or getting a disease?

____

Extra page for Extended family and Household

Relationship	Initials	Age	Gender	Occupation	Time spent with person on a daily or weekly basis	Contact level with link (person)<br>1:close<br>2:talking<br>3:touching<br>4:kissing

E. Survey questionnaire (filled in example)

Social Contact Survey Questionnaire:

Class: English 12

Household members:

Relationship	Initials	Age	Gender	Occupation or grade	Time spent with person on a daily basis (weekday)	Contact level with link (person) 1:close 2:talking 3:touching 4:kissing	Shared room? (yes/no)
Yourself	LG	17	Female	12th grade	*Na*	*Na*	no
Father	RG	50	Male	Scientist/ office work	2 hours	3	No
Mother	JG	51	Female	Teacher	2 hours	3	No
Sister	SG	15	Female	10th grade	2 hours	3	No

Do you spend additional time with household members during the weekend? If so, how much?	Yes, perhaps an extra 4 hours/ weekend

Extended Family members visited regularly (non household): group by their household

Relationship	Initials	Age	Gender	Occupation	Time spent with person on a weekly basis	Contact level with link (person) 1:close 2:talking 3:touching 4:kissing
None						

**Group Name**	**Time in group per day (weekday)**	**Size (number of people in group)**	**Age range in group**	**Number of primary links (people) in your group and initials of links, include teacher or coach if applicable**		**Relationship with primary links F: Family f: Friend A: Authority a: Acquaintance**	**Contact Level with primary links 1:close 2:talking 3:touching 4:kissing**	**Ages or age range for primary links**
School								
Period 0 none	50 min							
Period 1 none	50 min							
Period 2 Creative Writ.	50 min	25	15-18	4	LD ED MB P	f f A a	3 2 2 2	17 16 53 16
Period 3 English 12 AP	50 min	20	17-18	4	RH SD AV MB	f f f A	3 3 3 2	17 17 18 53
Period 4 Ind. Study	50 min	10	14-18	5	RH MH JC EG WV	f A f f f	2 2 2 3 2	17 50 16 16 18
Period 5 Physics	50 min	20	16-18	9	MG BN AV SD MT MD JS KG SS	f f f f A f f f a	3 3 2 2 2 2 3 3 2	17 17 18 17 40 17 17 17 17
Period 6 Physics	50 min	20	16-18	9	MG BN AV SD MT MD JS KG SS	f f f f A f f f a	3 3 2 2 2 2 3 3 2	17 17 18 17 40 17 17 17 17
Lunch	40 min	400	14-18	3	JC MG LS	16 17 17	2 3 3	16 17 17

Group Name	Time in group per week (include weekend)	Size (number of people in group)	Age range in group	Number of primary links (people) in your group and initials of links, include teacher or coach if applicable		Relationship with primary links F: Family f: Friend A: Authority a: Acquaintance	Contact Level with primary links 1:close 2:talking 3:touching 4:kissing	Ages or age range for primary links
School Clubs								
MESA	45 minutes	50	14-18	5	AV MA MP JS RB	f A A f f	3 2 2 3 2	18 38 35 17 17
Honor Society	30 minutes	100	15-18	3	MG EW AV	f a f	3 2 2	17 17 18
Non school Clubs/lessons/ etc.								
none								

Group Name	Time in group per week (include weekend)	Size (number of people in group)	Age range in group	Number of primary links (people) in your group and initials of links, include teacher or coach if applicable	Relationship with primary links<br>F: Family<br>f: Friend<br>A: Authority<br>a: Acquaintance	Contact Level with primary links<br>1:close<br>2:talking<br>3:touching<br>4:kissing	Ages or age range for primary links
Sports							
Cross Country Fall season (4 months)	14 hours	35	14-18	10 | JR SG CD CM<br>CW JS R EW D<br>MD	f F A A<br>A f f f f<br>f	3 3 2 2<br>2 3 3 3 3<br>2	17 15 55 45<br>58 17 16 17<br>17
Track and field Spring Season (4.5 months)	12 hours	50	14-18	7 | SG JR CM<br>HM CD MD<br>EW	F f A<br>F A f<br>a	3 3 2<br>3 2 2<br>2	15 17 55<br>17 45 17<br>17
Work							
Secretary, Health South Rehabilitation	4 hours	3	17-60	2 | SJ DG	a A	2 2	60 45
	xxxxxxxx	xxxxxxxx	xxxxx	xxxxxxxxxxxxxxxxx	Number of customers	Contact level	Age Range
Church							
Service	None						

Group Name	Time in group per week (include weekend)	Size (number of people in group)	Age range in group	Number of primary links (people) in your group and initials of links, include teacher or coach if applicable		Relationship with primary links F: Family f: Friend A: Authority a: Acquaintance	Contact Level with primary links 1:close 2:talking 3:touching 4:kissing	Ages or age range for primary links
Friend groups								
	4 hours	3	17-18	2	KK KG	f f	3 3	18 17
Neighborhood								
None								
Other								
None								

**Public Activity**	**Number of times per day, week, month, or year**	**How much time does this public activity take? (each time you do it)**	**Which *groups* do you do this public activity with?**	**How many *group members* do you have contacts with when you do this activity? (your primary links)**	**What is the range of level of contact with *group members* (primary links)?**	**Is this public activity already included in the time you spend in other groups?**	**How many <u>*non group members*</u> do you have contacts with when you do this public activity (random contacts)?**	**What is the range of level of contact with these <u>*non group members*</u> (random contacts)?**	**What is the age range of these <u>*non group members*</u> (random contacts)?**
Passing Periods, before-after school	5 per day	7 minutes	Friends, school classes	5	2-3	No	10-15	1, and 3	14-18
Car rides	2 per day	10 minutes	Friends, Household	0-3	1-3	No	0		
School bus	None								
City bus	None								
Mall	1 per month	30 minutes-1.5 hours	Friends, Household	0-3	1-3	No	5-20	1 and 3	7-50
Errands	3 per week	30 minutes to 1.5 hours	Alone, Household	0-2	1-3	No	2-6	1-2	15-55
Movies in theatres	1 every other month	3 hours	Friends, Household	1-4	1-3	No	5-10	1 and 3	15-55

**Public Activity**	**Number of times per day, week, month, or year**	**How much time does this public activity take? (each time you do it)**	**Which groups do you do this public activity with?**	**How many** *group members* **do you have contacts with when you do this activity? (your primary links)**	**What is the range of level of contact with** *group members* **(primary links)?**	**Is this public activity already included in the time you spend in other groups?**	**How many** *non group members* **do you have contacts with when you do this public activity (random contacts)?**	**What is the range of level of contact with these** *non group members* **(random contacts)?**	**What is the age range of these** *non group members* **(random contacts)?**
Concerts	none								
Sport/game event participant	1 per week	4 hours	Sports	15	1-3	Yes	10-30	1-3	14-55
Sport/game event attendance	none								
Dances	3 per year	4 hours	Friends, School	8-12	1-3	No	20-50	1-3	14-18
Parties	none								
Eating out	1 every other month	1-2 hours	Friends, Household	1-5	1-3	No	3-7	1-2	14-55

Amount of time spent per day doing things on your own (homework, TV, reading, etc.)	5 hours
